# Trazodone Overdose Manifesting as Hypotension and QT Prolongation

**DOI:** 10.7759/cureus.36871

**Published:** 2023-03-29

**Authors:** Gaurav Mohan, Ashika Ajitkumar, Poorva Bhide, Jayashree Ravilla, Violet Kramer

**Affiliations:** 1 Internal Medicine, Monmouth Medical Center, Long Branch, USA; 2 Internal Medicine, Ascension Saint Agnes Hospital, Baltimore, USA; 3 Pulmonary and Critical Care, Monmouth Medical Center, Long Branch, USA

**Keywords:** medical toxicology, drug-induced hypotension, drug-induced qtc prolongation, toxicity, trazodone

## Abstract

Trazodone is a medication used to treat major depressive disorder (MDD). It is in the serotonin-antagonist-and-reuptake-inhibitor class of medications with anti-cholinergic effects. Trazodone is known for its sedative effects and is hence often prescribed in those with MDD with concurrent insomnia. While few, there have been reports of patients overdosing on trazodone and developing QTc prolongation leading to fatal arrhythmias such as torsades des pointes and variable atrioventricular blocks. We present a case of a 45-year-old female with a past medical history of MDD and anxiety, who presented with dizziness, transient ataxia, and urinary incontinence following ingestion of five 100 mg trazodone tablets. Although her vitals were initially stable on admission, her EKG was concerning for QTc prolongation of 502 ms. A few hours later, she started developing hypotension and progressive QTc prolongation, with a peak of 586 ms. Given the high risk of decompensation, the patient was admitted to the ICU for further care where she received adequate supportive management in the form of fluid resuscitation, electrolyte repletion, serial EKGs every hour, and telemetry monitoring for arrhythmias, with eventual improvement in her clinical condition. Trazodone poisoning, while rare, can be fatal and hence requires close monitoring to prevent complications. Clinicians must be aware of these possible adverse outcomes when managing trazodone toxicity.

## Introduction

Trazodone is a relatively safe antidepressant that blocks the histamine and alpha-1-adrenergic receptors. It also inhibits serotonin type 2 receptors and serotonin transporter. It is an FDA-approved anti-depressant with off-label use for sleep induction for those with or without depression, anxiety, substance abuse, bulimia, and even post-traumatic stress disorder. Standard dosing varies between 75 mg and 400 mg per day. Trazodone has a lower anticholinergic effect than other antidepressants and hence has lower arrhythmogenic effects. Other signs of trazodone toxicity are anticholinergic symptoms such as dry mouth, ataxia, nausea, vomiting, dizziness, urinary incontinence, hypotension, priapism, seizures, and rarely serotonin syndrome [1]. While few, there have been reports of patients overdosing on trazodone and developing QTc prolongation leading to fatal arrhythmias such as torsades des pointes and variable atrioventricular (AV) blocks [2-4]. Our patient additionally presented with hypotension, requiring close monitoring in the intensive care unit.

## Case presentation

**Figure 1:**
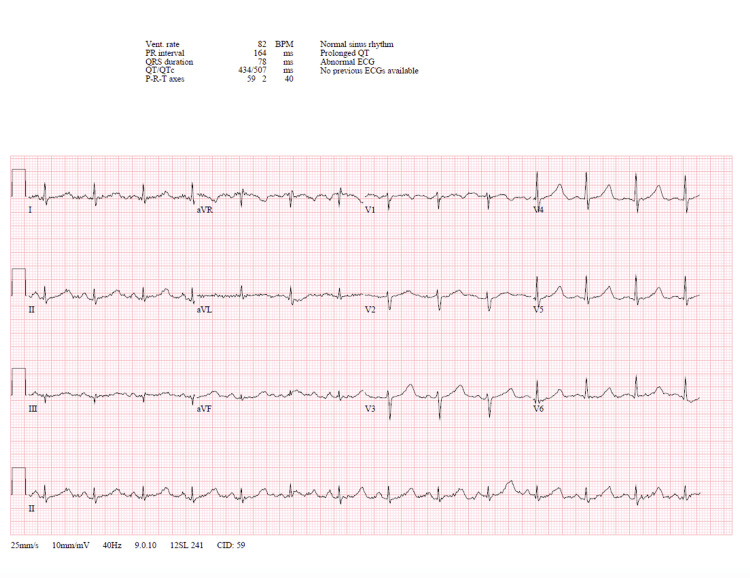
EKG on admission showing normal sinus rhythm with a heart rate of 82 beats per minute and a prolonged QTc of 507 ms

Our patient is a 45-year-old woman with a past medical history of MDD and anxiety, who presented to the emergency department with a two-hour history of ataxia, dizziness, and urinary incontinence. She had ingested five 100 mg pills of trazodone two hours prior to the onset of symptoms. After a family altercation, she took the medications to help her calm down and sleep. Her only other medications were bupropion and clonazepam, none of which she had consumed in the prior week. She has no previous history of cardiac arrhythmias or prolonged QTc, and neither does she have a family history of the same. On presentation, vital signs revealed a blood pressure of 130/76 mm of Hg, a heart rate of 88 beats per minute, and a respiratory rate of 14 breaths per minute. Physical examination was unremarkable. We do not have a baseline EKG for this patient. Her EKG from admission revealed a heart rate of 82 beats per minute in normal sinus rhythm and a prolonged QTc interval of 502 ms, as shown below in Figure 1.

**Figure 2:**
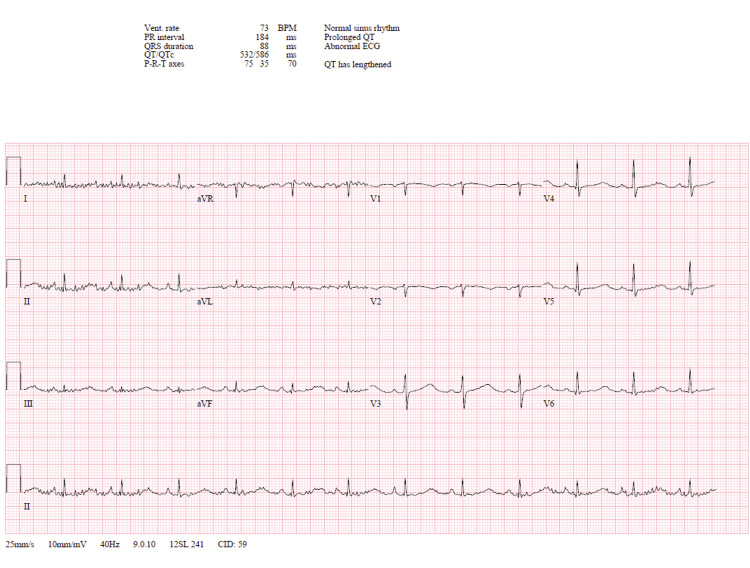
EKG depicting the patient's highest recorded QTc during her hospital course, with a heart rate of 73 beats per minute and QTc of 586 ms

Initial laboratory workup revealed a hemoglobin of 11.8 g/dL, a white blood cell count of 7700 per microliter, and a platelet count of 298,000 per microliter. The basic chemistry panel revealed the following: sodium 142 mmol/L, potassium 3.7 mmol/L, chloride level of 109 mmol/L, bicarbonate 28 mmol/L, blood urea nitrogen 9 mg/dL, creatinine 0.7 mg/dL, anion gap of 7, and glucose 89 mg/dL. Urinalysis was unremarkable. Serum acetaminophen, ethanol, and salicylate levels were negative. A comprehensive urine toxicology screen (done through gas chromatography) was also negative; however, it does not test for trazodone. Unfortunately, we could not obtain a serum trazodone level either due to laboratory limitations. While the patient initially insisted on being discharged, serial EKGs showed a progressive prolongation of the QTc interval to 586 ms over five hours as depicted in Figure 2. She was eventually admitted to the ICU to continuously monitor her vitals and QTc interval.

**Figure 3:**
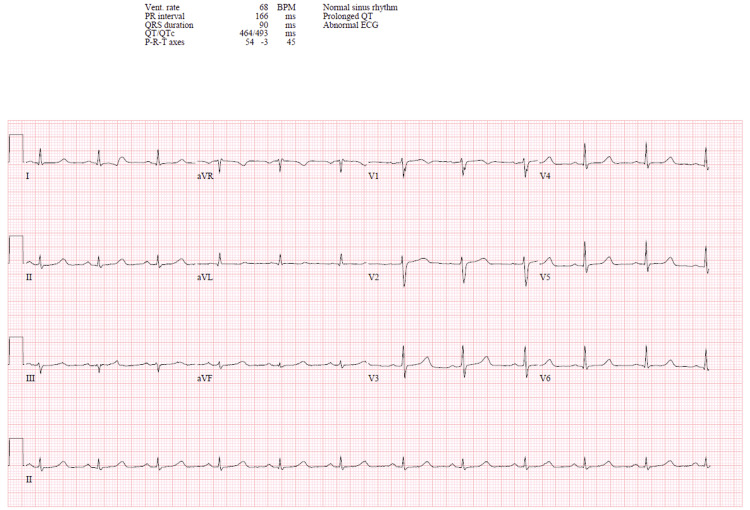
EKG depicting this patient's improved QTc interval, with a heart rate of 68 beats per minute and QTc of 493 ms

While in the ICU, the patient also developed hypotension for a period of five hours on the day of admission with her blood pressure ranging between 82/47 mm of Hg and 100/51 mm of Hg. While we initially anticipated the need for pressors, her blood pressure began improving with intravenous fluid boluses alone. There were no cardiac arrhythmias noted on the telemetry review. Hourly EKGs were obtained with an eventual improvement of the QTc to 493 ms, observed about 36 hours after the highest QTc was recorded, as depicted in Figure 3. Electrolytes were regularly monitored and were noted to be within normal range throughout her hospital course. Given that her QTc had improved and hypotension had resolved, the patient was downgraded from the ICU to a regular medical unit prior to being discharged home.

## Discussion

What makes our patient unique is the presence of hypotension requiring fluid boluses and ICU monitoring of a prolonged QTc interval. A retrospective review done examining over 118,000 single-substance trazodone exposures reported to US poison centers revealed that only 12% of patients with reported outcomes had QTc prolongation, and only 7% of patients had hypotension, making our patient’s clinical presentation extremely unique [5]. However, the true number of fatalities from trazodone overdose is not known. Peak plasma concentrations of trazodone occur approximately one hour after oral administration when taken on an empty stomach or two hours after oral administration when taken with food. Plasma concentrations of trazodone decline in a biphasic manner. The half-life of trazodone in the initial phase is about three to six hours, and the half-life in the terminal phase is about five to nine hours. The therapeutic range for plasma trazodone concentrations and the relationship of plasma concentrations to clinical response and toxicity has not been established [6]. This patient also reports taking bupropion, although she had reported not taking this medication for about one week prior to her presentation. Bupropion is not known to cause QTc prolongations, and neither are there any reported interactions between bupropion and trazodone. Although our patient appeared clinically well on presentation and was requesting to be sent home, she developed progressively worsening QTc prolongation and clinically significant hypotension. We also recognize that due to laboratory limitations, we were unable to obtain a serum trazodone level. However, given the fact that the patient's presentation was consistent with that of trazodone toxicity and did improve over a period of time with supportive measures, we believed that reporting this case would be a good learning tool for clinicians. This will also encourage clinicians to have a low threshold for closely monitoring patients with suspected trazodone toxicity. As there is no antidote available against trazodone, management is largely supportive. It is also important to maintain optimal electrolyte levels and contact the local department of toxicology to report an overdose and receive updated guidelines regarding management.

## Conclusions

Trazodone, with its good safety profile, is seldom known to cause major side effects. Most of the cases of trazodone overdose have occurred concomitantly with other agents. However, as illustrated above, overdose can cause a wide range of critical conditions that can be fatal. In our patient, close monitoring of her vitals on telemetry and hourly EKGs in the ICU was essential to manage her safely to ensure that her vitals were stable and that she did not develop any fatal arrhythmias. Clinicians must be aware of these possible adverse outcomes when managing trazodone toxicity.
